# Supplementary material for: Quantitative Comparison of Statistical Methods for Analyzing Human Metabolomics Data
Source: Metabolites. 2022 Jun 4;12(6):519. doi: 10.3390/metabo12060519 (PMC9227835; doi:10.3390/metabo12060519)

SUPPLEMENTAL MATERIAL

Table S1. Known Metabolite Compounds Assayed by the LC-MS Method Used

Metabolite Type	Mass-to-Charge Ratio	ID
polar molecule	227.0645	DEOXYURIDINE
free fatty acid	227.2012	Myristic Acid
polar molecule	241.0738	LUMICHROME
free fatty acid	241.2170	Pentadecanoic Acid
polar molecule	242.0801	CYTIDINE
free fatty acid	253.2172	Palmitoleic Acid
free fatty acid	255.2329	Palmitic Acid
eicosanoid	265.1812	tetranor 12(R) HETE
polar molecule	267.0475	HOMOCYSTINE
free fatty acid	267.2330	Heptadecaenoic Acid
steroid	269.1758	Estrone
free fatty acid	275.2020	Stearidonic Acid
free fatty acid	277.2175	GAMMA-LINOLENATE
free fatty acid	279.2331	LINOLEATE
free fatty acid	281.2489	ELAIDATE
eicosanoid	291.1967	13S-HpOTrE(gamma)
eicosanoid	293.2122	13-oxoODE
eicosanoid	293.2122	9-oxoODE
eicosanoid	295.2275	9-HODE
eicosanoid	299.2010	15-oxoETE
eicosanoid	299.2039	5-oxoETE
free fatty acid	301.2172	Eicosapentaenoic Acid
free fatty acid	303.2331	Arachidonic Acid
free fatty acid	305.2486	Eicosatrienoic Acid
free fatty acid	307.2644	Eicosadienoic Acid
free fatty acid	309.2044	MYRISTATE
free fatty acid	309.2798	Gondolic Acid
free fatty acid	311.2955	ARACHIDATE
free fatty acid	313.2387	PALMITOLEATE

eicosanoid	315.1951	15-keto-PGA1
eicosanoid	315.1964	13(S) HOTrE(y)
eicosanoid	315.1971	bicyclo PGE2
eicosanoid	315.2000	5S-HpEPE
eicosanoid	315.2000	15d PGJ2
eicosanoid	317.2110	HXA3
eicosanoid	317.2115	5(S) HEPE
eicosanoid	317.2117	18(S) HEPE
eicosanoid	317.2118	15(S) HEPE
eicosanoid	317.2121	14(15) EpETE
eicosanoid	317.2128	12epi LTB4
eicosanoid	317.2136	5,15-diHETE
eicosanoid	319.2260	16-HETE
eicosanoid	319.2278	11-HETE
eicosanoid	319.2283	14,15-EET
eicosanoid	319.2291	5,6-EET
eicosanoid	321.1712	11-dehydro-2,3-dinor-TXB2
eicosanoid	321.2423	8(S) HETrE
eicosanoid	321.2435	15(S) HETrE
polar molecule	323.0974	CELLOBIOSE
endocannabinoid	326.3038	Stearoyl EA
free fatty acid	327.2326	Docosahexaenoic Acid
polar molecule	329.0161	DEOXYURIDINE-MONOPHOSPHATE
eicosanoid	331.1890	9S-HpOTrE
steroid	331.1909	Estradiol
eicosanoid	331.1916	PGD3
eicosanoid	333.2061	8-iso-15-keto-PGF2alpha
eicosanoid	333.2064	PGA2
eicosanoid	333.2070	12oxo LTB4
eicosanoid	333.2070	dhk PGE2
eicosanoid	333.2071	13,14-dihydro-15-keto-PGA2
eicosanoid	333.2072	ent-PGE2
eicosanoid	333.2074	LXB4
eicosanoid	333.2077	20cooh AA

eicosanoid	333.2080	8-iso-PGA2
free fatty acid	333.2811	Docosatrienoic Acid
eicosanoid	335.2222	15S-HpETE
eicosanoid	335.2226	PGF2beta
eicosanoid	335.2228	8,12-iso-iPF2Ã -VI-1,5-lactone
eicosanoid	335.2228	14,15-DiHETE
eicosanoid	335.2228	8-iso-PGA1
eicosanoid	335.2232	15R-PGE1
eicosanoid	335.2234	15R-PGF2alpha
eicosanoid	335.2249	5,6-diHETE
free fatty acid	335.2959	Docosadienoic Acid
free fatty acid	337.3116	ERUCATE
eicosanoid	339.2175	12-HHTrE
free fatty acid	339.2533	Linoleic Acid
free fatty acid	339.3269	Behenic Acid
eicosanoid	341.2163	17S-HpDHA
free fatty acid	343.2853	Stearic Acid
eicosanoid	351.2170	9-oxoOTrE
eicosanoid	351.2202	8-iso-PGE2
free fatty acid	351.3270	Tricosenoic Acid
eicosanoid	353.2319	13(S) HOTrE
eicosanoid	353.2337	9(S) HOTrE
eicosanoid	353.2343	11b dhk PGF2a
free fatty acid	353.3427	TRICOSANOATE
eicosanoid	355.2486	13-HODE
eicosanoid	355.2486	12,13 EpOME
endocannabinoid	358.2962	Palmitoyl Ethanolamide
endocannabinoid	358.2966	Palmitoyl EA or
steroid	359.1867	Cortisone
eicosanoid	359.2222	5,6-diHETrE
free fatty acid	359.2971	Docosaenoic Acid
eicosanoid	363.2556	1a,1b-dihomo-PGE1
eicosanoid	363.2557	dihomo PGF2a
polar molecule	364.0595	CYTIDINE 2',3'-CYCLIC PHOSPHATE

free fatty acid	365.2658	STEARATE
free fatty acid	367.3583	Lignoceric Acid
bile acid	375.2906	Lithocholic Acid
eicosanoid	379.2486	15-HETE
endocannabinoid	382.2975	Linoleoyl EA
endocannabinoid	384.3114	Oleoyl Ethanolamide
endocannabinoid	384.3128	Oleoyl EA
free fatty acid	387.3286	Nervonic Acid
steroid	389.2309	11-deoxycortisosterone or 17a-hydroxyprogesterone
bile acid	389.2697	Cholic Acid
free fatty acid	391.2850	Adrenic Acid
bile acid	391.2862	Deoxycholic Acid
bile acid	391.2863	Ursodeoxycholic acid
eicosanoid	393.2280	15d PGD2
eicosanoid	393.2294	PGB2
eicosanoid	395.2429	LTB4
eicosanoid	395.2433	15-epi-PGA1
eicosanoid	395.2442	PGA1
eicosanoid	395.2445	12,13 diHOME
polar molecule	397.3365	ERUCATE
polar molecule	401.1297	PALATINOSE
eicosanoid	403.2485	15 oxoEDE
eicosanoid	403.2500	14 HDoHE
steroid	405.2265	Unk
steroid	405.2271	Unk
steroid	405.2277	11-deoxycortisol
steroid	405.2287	Unk
steroid	405.2295	CORTEXOLONE
polar molecule	407.0623	INOSINE MONOPHOSPHATE
bile acid	407.2804	b-Muricholic Acid
eicosanoid	411.2365	6S-LXA4
endocannabinoid	415.3076	OLEOYL-GLYCEROL
eicosanoid	417.2246	12S-HpETE

eicosanoid	417.2284	8,15-diHETE
steroid	419.2078	CORTISONE
steroid	421.2239	CORTISOL
steroid	421.2241	Cortisol
endocannabinoid	423.3101	2-AG ether
steroid	425.2547	allo-Tetrahydrocortisol
free fatty acid	425.3617	NERVONATE
bile acid	430.2965	Glycodeoxycholic Acid
endocannabinoid	430.3028	Docosahexanoyl EA
bile acid	435.3122	LITHOCHOLATE
endocannabinoid	437.2899	2-AG maybe
bile acid	448.3074	Glycoursodeoxycholic Acid
eicosanoid	451.2348	6k PGF1a
bile acid	451.3071	Chenodeoxycholic acid
bile acid	464.3021	GLYCOCHOLATE
bile acid	470.2848	Glycochenodeoxycholic Acid
bile acid	480.2745	Tauroursodeoxycholic acid
eicosanoid	495.2607	14,15 LTD4
bile acid	498.2904	Taurodeoxycholic Acid
bile acid	514.2843	Taurocholic Acid
bile acid	520.2656	Taurochenodeoxycholic acid
polar molecule	540.0464	ADENOSINE DIPHOSPHATE RIBOSE
polar molecule	610.0507	ADP-GLUCOSE

Figure S1. For analyses of continuous outcome (Panel A) and binary outcome (Panel B), the sensitivity, specificity, positive predictive value (PPV), negative predictive value (NPV), and false positive rate are displayed (as percent color fill of each bar) for each statistical method, reflecting their ability to correctly identify the top ten simulated metabolite associations, across varying numbers of total metabolite measures (M=200, or M=2000) in smaller-sized study samples collected from varying numbers of study subjects (N=50, N=100, N=200, N=1000, or N=5000). PCR, principal components regression; BON, Bonferroni; FDR, false discovery rate; LASSO, lease absolute shrinkage and selection operator; SPLS, sparse partial least squares; RF, random forests.

A.

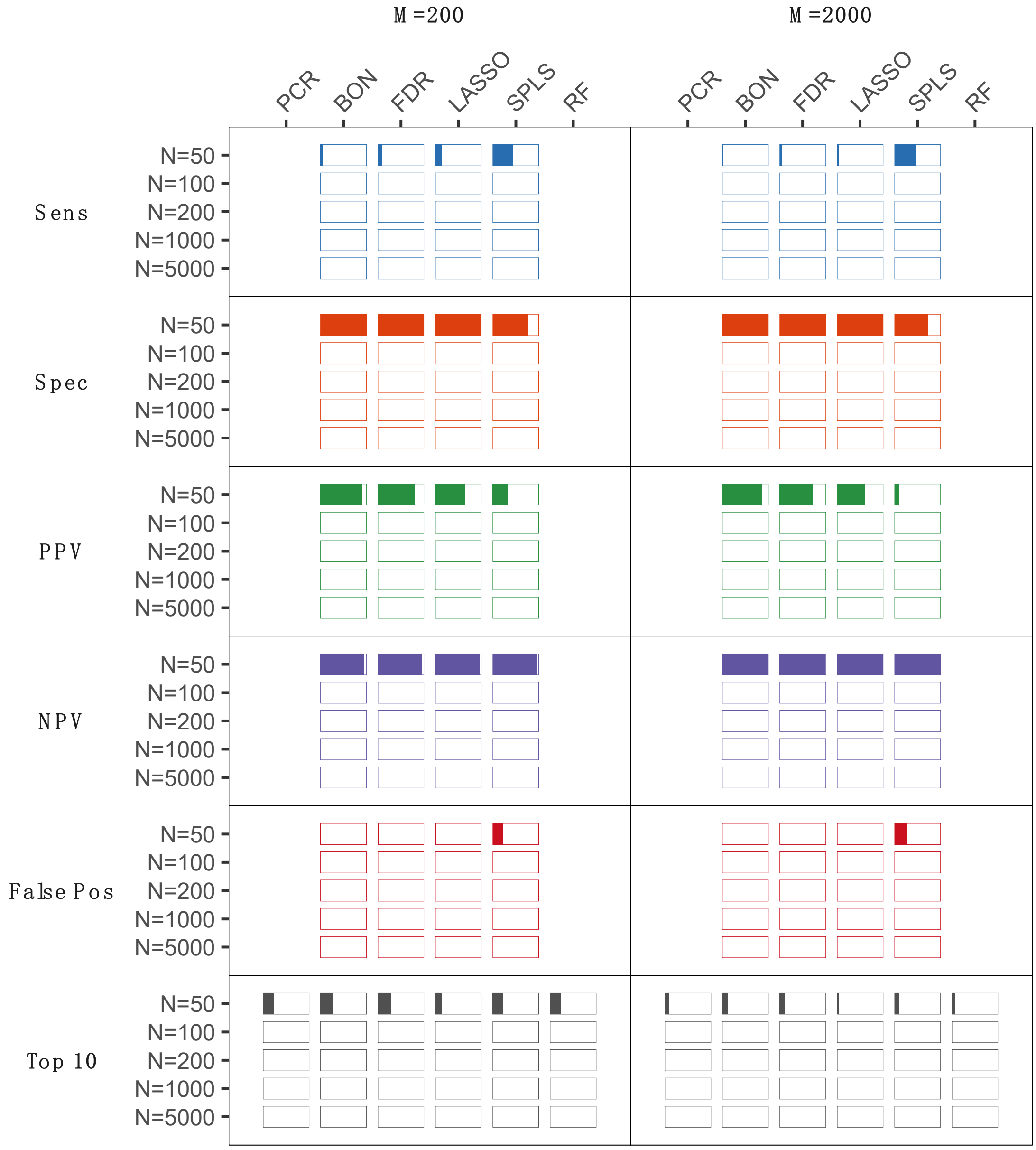

B.

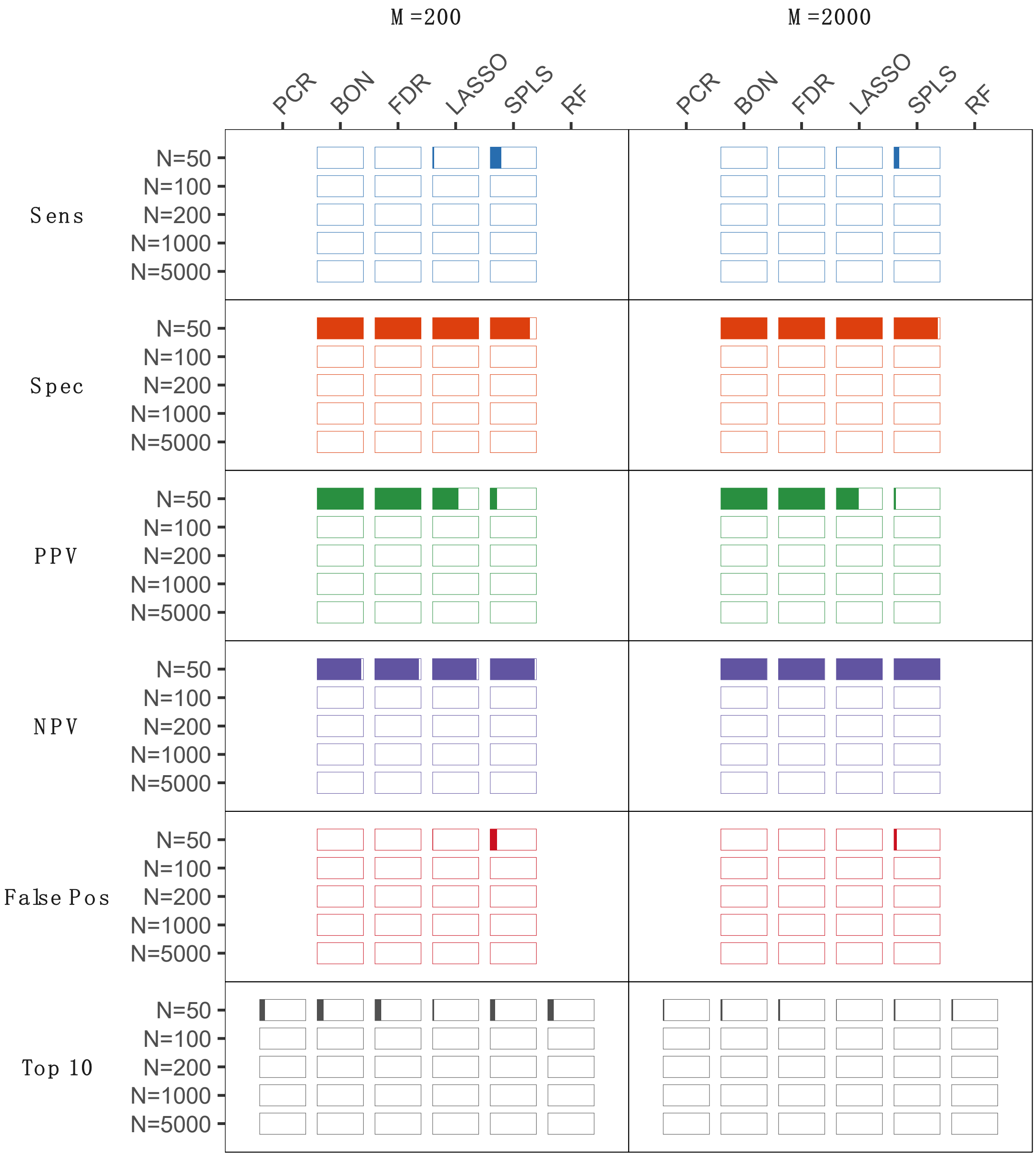
M =200
M =2000
PCR
BON
FDR
LASSO
SPLS
RF
Sens
Spec
PPV
NPV
False Pos
Top 10
N=50
N=100
N=200
N=1000
N=5000

Figure S2. Estimates of power to detect metabolites associated with a continuous outcome for a given effect size based on simulations. The estimated power to detect the top ten metabolite associations per effect size is shown for each statistical method, across varying numbers of total metabolite measures (M=200, or M=2000) in study samples collected from varying numbers of study subjects (N=200, N=1000, or N=5000). BON, Bonferroni; FDR, false discovery rate; LASSO, lease absolute shrinkage and selection operator; SPLS, sparse partial least squares.

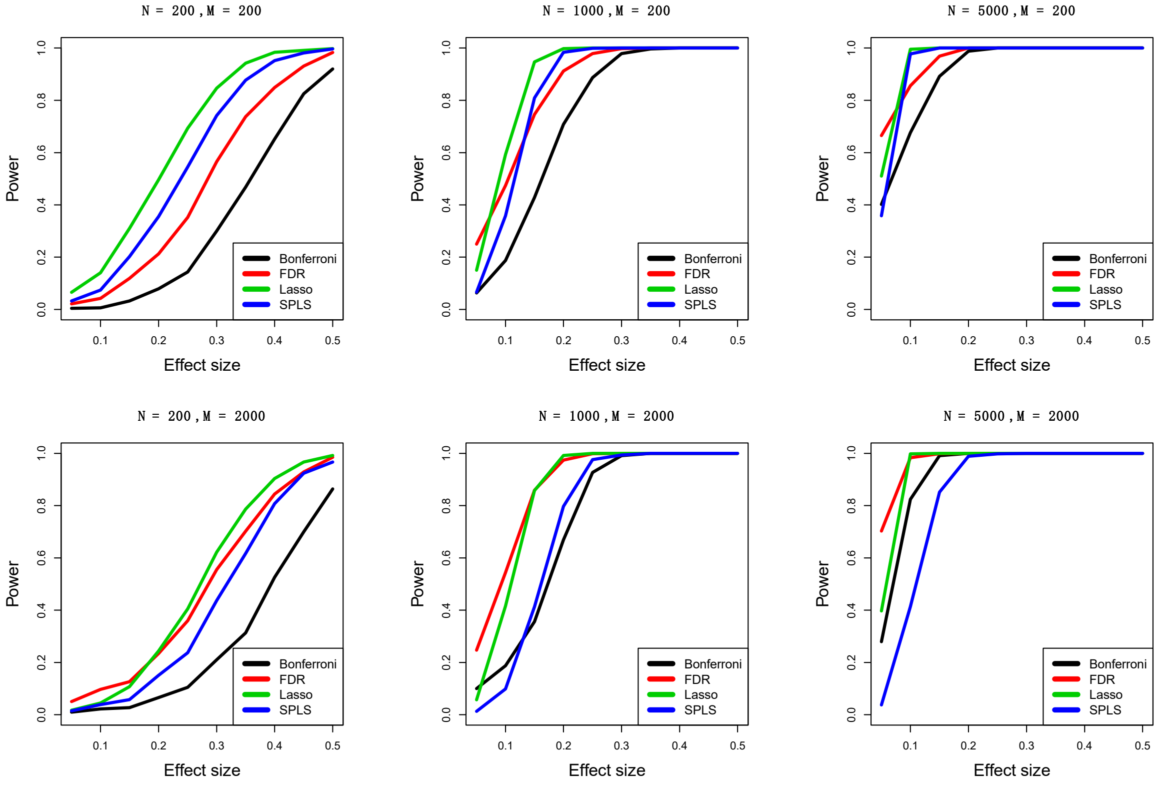

Figure S3. Estimates of power to detect metabolites associated with a binary outcome for a given effect size based on simulations. The estimated power to detect the top ten metabolite associations per effect size is shown for each statistical method, across varying numbers of total metabolite measures (M=200, or M=2000) in study samples collected from varying numbers of study subjects (N=200, N=1000, or N=5000). BON, Bonferroni; FDR, false discovery rate; LASSO, lease absolute shrinkage and selection operator; SPLS, sparse partial least squares.

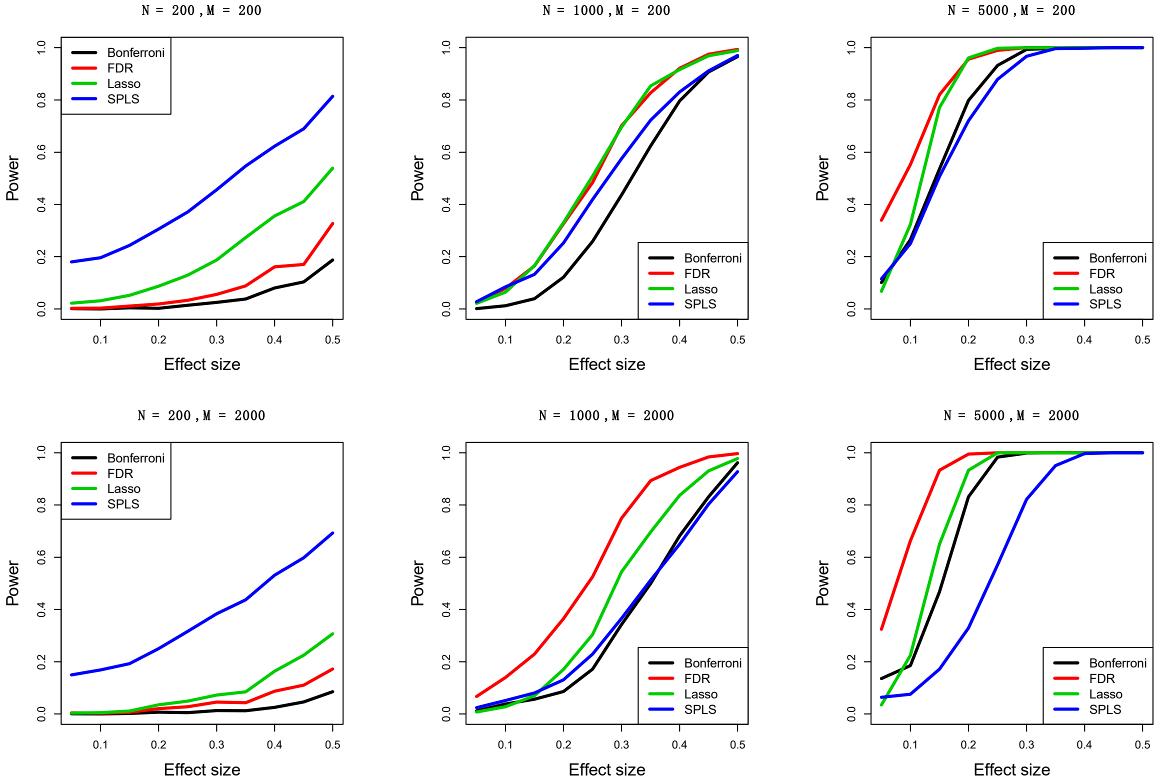

Figure S4. Results for a continuous outcome based on simulations with positive and negative inter-metabolite correlations. The sensitivity, specificity, positive predictive value (PPV), negative predictive value (PPV), and false positive rate are displayed (as percent color fill of each bar) for each statistical method, reflecting their ability to correctly identify the top ten simulated metabolite associations, across varying numbers of total metabolite measures (M=200, or M=2000) in study samples collected from varying numbers of study subjects (N=200, N=1000, or N=5000). PCR, principal components regression; BON, Bonferroni; FDR, false discovery rate; LASSO, lease absolute shrinkage and selection operator; SPLS, sparse partial least squares; RF, random forests.

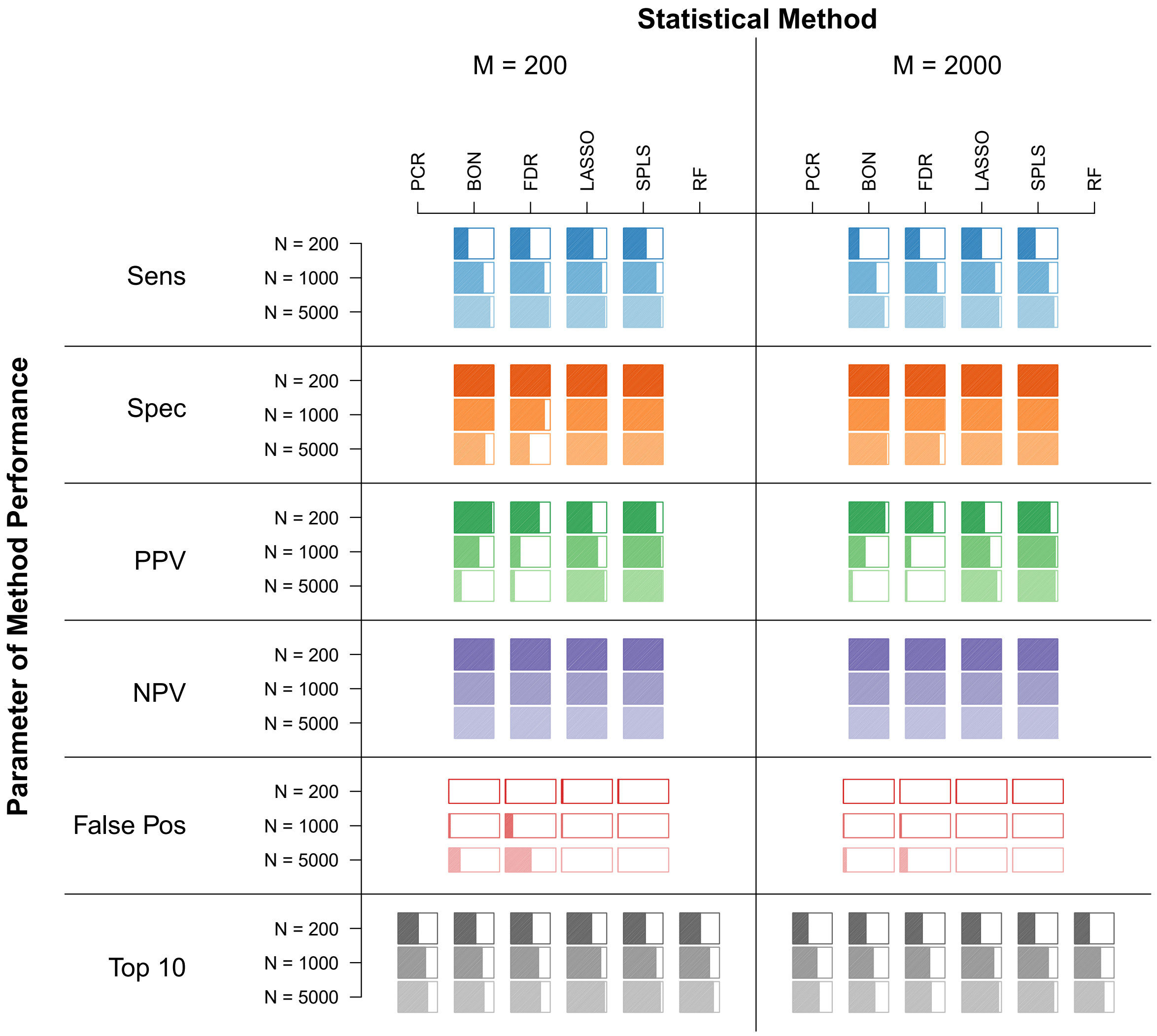

Figure S5. Results for a binary outcome based on simulations with positive and negative inter-metabolite correlations. The sensitivity, specificity, positive predictive value (PPV), negative predictive value (PPV), and false positive rate are displayed (as percent color fill of each bar) for each statistical method, reflecting their ability to correctly identify the top ten simulated metabolite associations, across varying numbers of total metabolite measures (M=200, or M=2000) in study samples collected from varying numbers of study subjects (N=200, N=1000, or N=5000). PCR, principal components regression; BON, Bonferroni; FDR, false discovery rate; LASSO, lease absolute shrinkage and selection operator; SPLS, sparse partial least squares; RF, random forests.

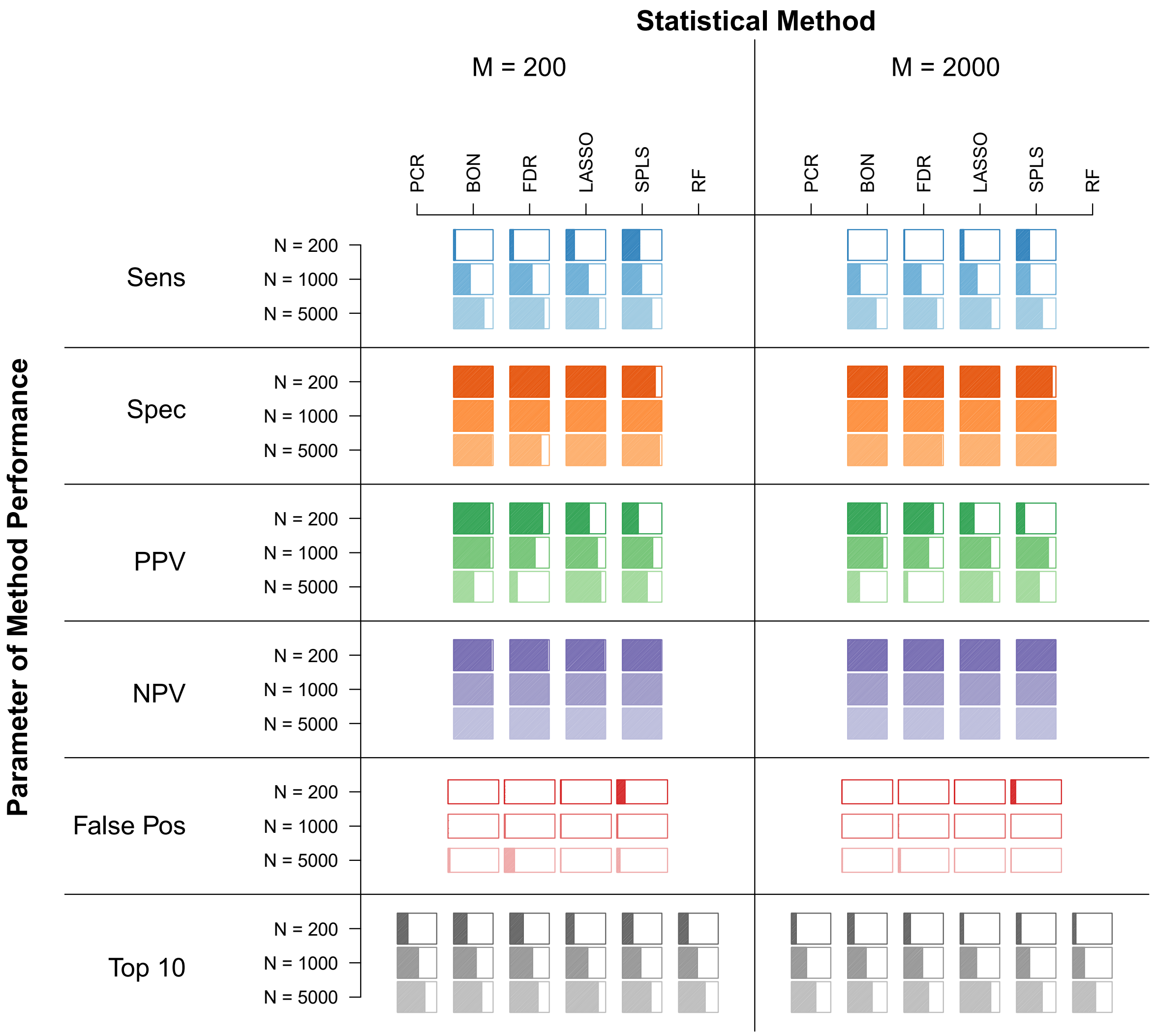

Figure S6. Results for a continuous outcome based on simulations with highly correlated important covariates. The sensitivity, specificity, positive predictive value (PPV), negative predictive value (PPV), and false positive rate are displayed (as percent color fill of each bar) for each statistical method, reflecting their ability to correctly identify the top ten simulated metabolite associations, across varying numbers of total metabolite measures (M=200, or M=2000) in study samples collected from varying numbers of study subjects (N=200, N=1000, or N=5000). PCR, principal components regression; BON, Bonferroni; FDR, false discovery rate; LASSO, lease absolute shrinkage and selection operator; SPLS, sparse partial least squares; RF, random forests.

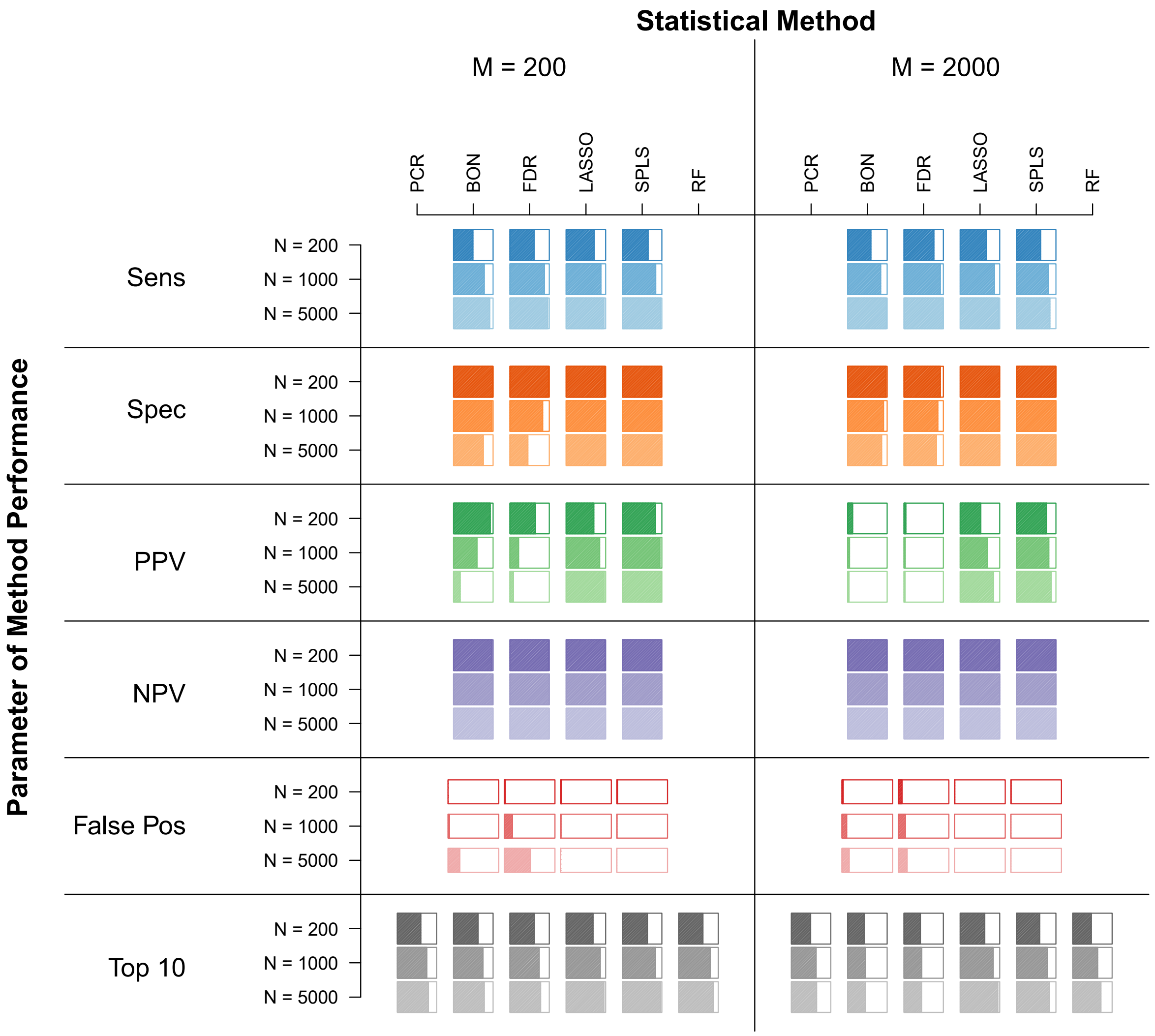

Figure S7. Results for a binary outcome based on simulations with highly correlated important covariates. The sensitivity, specificity, positive predictive value (PPV), negative predictive value (PPV), and false positive rate are displayed (as percent color fill of each bar) for each statistical method, reflecting their ability to correctly identify the top ten simulated metabolite associations, across varying numbers of total metabolite measures (M=200, or M=2000) in study samples collected from varying numbers of study subjects (N=200, N=1000, or N=5000). PCR, principal components regression; BON, Bonferroni; FDR, false discovery rate; LASSO, lease absolute shrinkage and selection operator; SPLS, sparse partial least squares; RF, random forests.

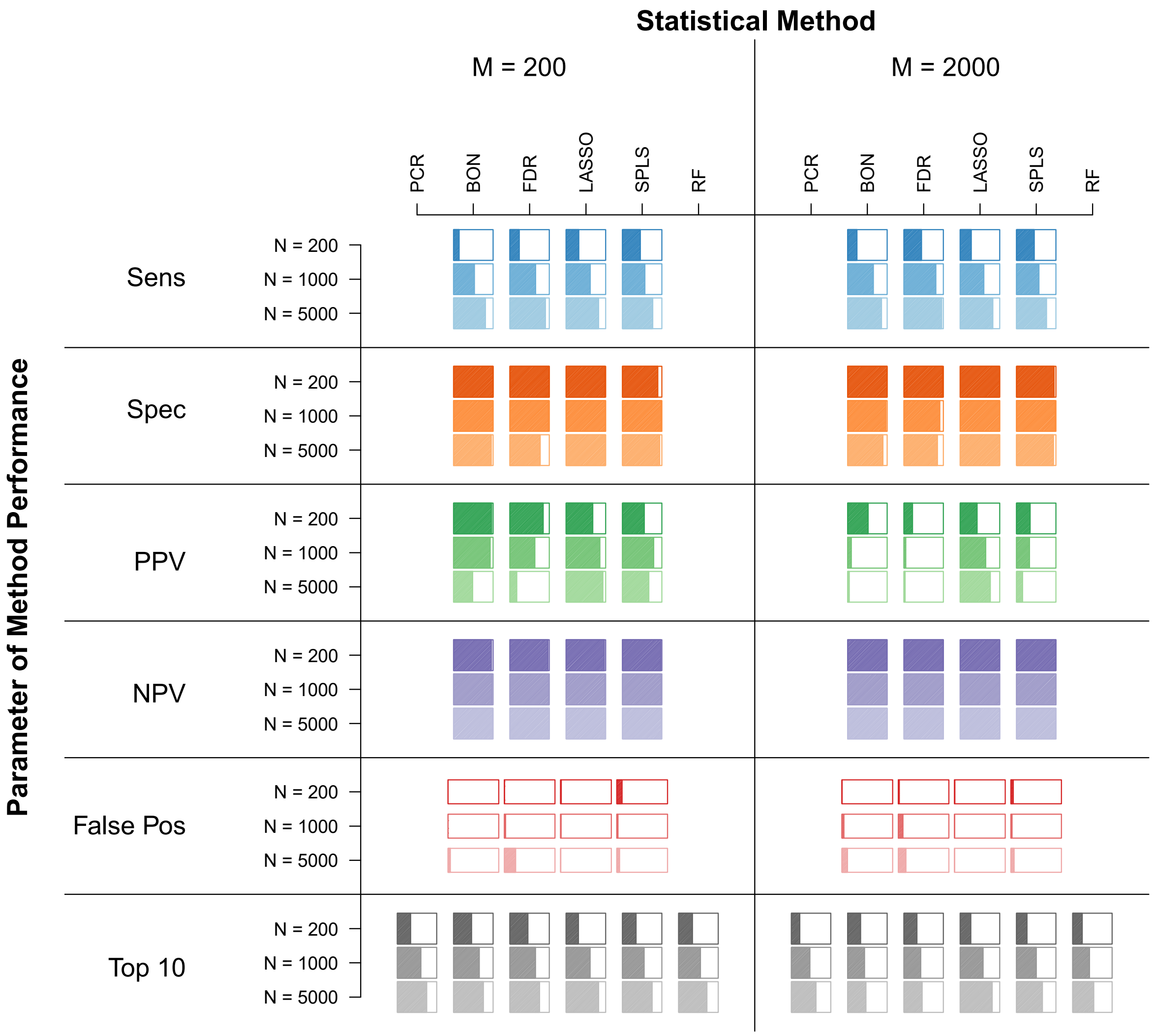

Figure S8. Results of analyzing actual, experimentally derived metabolomics data while controlling for batch effects. The number of metabolites found in association with age (continuous outcome) and sex (binary outcome) from experimentally derived metabolomics studies (see text) for different statistical methods applied: false discovery rate (FDR), sparse partial least squares (SPLS), and least absolute shrinkage and selection operator (LASSO). The number of metabolite correlates found in common by the different methods is relatively small compared to the total number of apparently significantly associated metabolites.

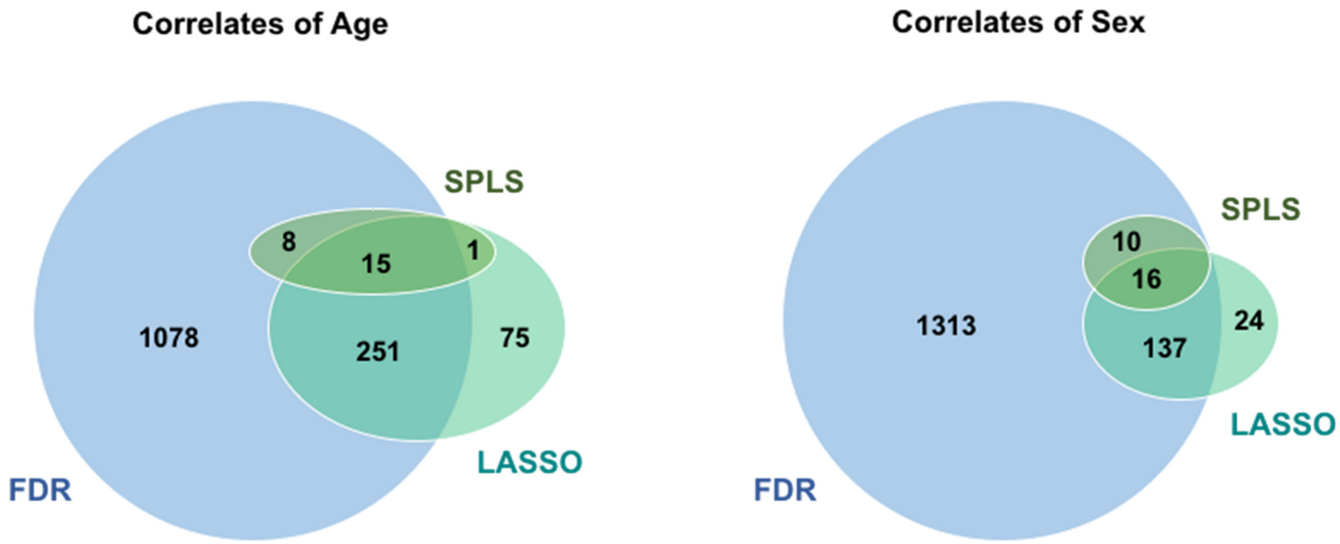

Supplement: Supplementary file 1 [file metabolites-12-00519-s001.zip › metabolites-1605975-supplementary.pdf]
